# Identification of Small Molecule Enhancers of Immunotherapy for Melanoma

**DOI:** 10.1038/s41598-020-62369-1

**Published:** 2020-03-30

**Authors:** Christopher Dextras, Myagmarjav Dashnyam, Lesley A. Mathews Griner, Janani Sundaresan, Bryan Chim, Zhiya Yu, Suman Vodnala, Chyi-Chia Richard Lee, Xin Hu, Noel Southall, Juan J. Marugan, Ajit Jadhav, Nicholas P. Restifo, Nicolas Acquavella, Marc Ferrer, Anju Singh

**Affiliations:** 1 Division of Preclinical Innovation, National Center for Advancing Translational Sciences (NCATS), National Institutes of Health, Rockville, Maryland USA; 2 Laboratory of Immune System Biology, National Institute of Allergy and Infectious Diseases, NIH, Bethesda, Maryland USA; 3 Center for Cancer Research, National Cancer Institute (NCI), NIH, Bethesda, Maryland USA

**Keywords:** Melanoma, High-throughput screening, Translational immunology

## Abstract

Small molecule based targeted therapies for the treatment of metastatic melanoma hold promise but responses are often not durable, and tumors frequently relapse. Response to adoptive cell transfer (ACT)-based immunotherapy in melanoma patients are durable but patients develop resistance primarily due to loss of antigen expression. The combination of small molecules that sustain T cell effector function with ACT could lead to long lasting responses. Here, we have developed a novel co-culture cell-based high throughput assay system to identify compounds that could potentially synergize or enhance ACT-based immunotherapy of melanoma. A *BRAF*^*V600E*^ mutant melanoma cell line, SB-3123_p_ which is resistant to Pmel-1-directed ACT due to low gp100 expression levels was used to develop a homogenous time resolve fluorescence (HTRF), screening assay. This high throughput screening assay quantitates IFNγ released upon recognition of the SB-3123_p_ melanoma cells by Pmel-1 CD8^+^ T-cells. A focused collection of approximately 500 small molecules targeting a broad range of cellular mechanisms was screened, and four active compounds that increased melanoma antigen expression leading to enhanced IFNγ production were identified and their *in vitro* activity was validated. These four compounds may provide a basis for enhanced immune recognition and design of novel therapeutic approaches for patients with BRAF mutant melanoma resistant to ACT due to antigen downregulation.

## Introduction

Melanoma remains the most frequent cause of skin cancer related death in young Caucasian adults^1^. Incidence rates continue to increase for melanoma with 100,000 new cases diagnosed every year leading to 8000 deaths annually in the United States^2^. Metastatic melanoma has a poor prognosis, and 5‐year survival rates for individuals with regional and distant stage disease are 61.7% and 15.2%, respectively^3^. The development of novel therapies to target melanoma, specifically the metastatic form, is a great unmet medical need^4^.

Among metastatic melanomas, oncogenic BRAF mutations are found in over half of patients with a substitution of glutamic acid for valine at amino acid position 600 (V600E) of the BRAF kinase domain comprising the most common mutation, accounting for 70–88% of cases^5,6^. This mutation promotes aberrant cell growth through constitutive activation of the RAF-MEK1/2-ERK1/2-MAP (MAPK) pathway^5^. The second most prevalent BRAF mutation is V600K accounting for 5–30% of cases and studies have associated it with an increased risk for brain and lung metastases compared to patients harboring a *BRAF*^V600E^ mutation^7^. Inhibition of the BRAF V600E oncoprotein with the small molecule vemurafenib results in profound tumor regression in ≥50% of patients^7–9^. Vemurafenib has also extended survival in patients with the less common BRAF V600K mutation^7–9^. Patients treated with BRAF inhibitors alone or in combination with MEK inhibitors have shown high objective response rates but have limited therapeutic duration and melanomas recur in almost every patient due to the development of drug resistance^10,11^.

A therapeutic approach aimed at tackling metastatic and drug resistant disease is currently under investigation using ACT^12,13^. The process of ACT involves the isolation of autologous tumor-infiltrating lymphocytes (TILs) from a patient’s tumor mass by harvesting single-cell suspensions from tumor fragments, and expanding the isolated cells using interleukin-2, a known T-cell growth factor^14^. The cells are then infused back into the patient following a preparative lymphodepletion regimen. This process can produce profound and even ‘curative’ antitumor responses that can be durable due to lasting memory of the adaptive immune system. However, even in these patients, melanomas often acquire resistance to immune therapy leading to relapse.

Several studies have investigated the mechanism(s) for resistance to immunotherapy and have studied tumor cell intrinsic and extrinsic factors that contribute to inhibition of anti-tumor immune responses^15,16^. Besides altered signaling pathways, immuno-suppressive tumor microenvironment can lead to resistance to ACT. Melanoma cells are inherently plastic in nature and often de-differentiate in the inflammatory tumor microenvironment, lose antigen recognition, and are no longer recognized and killed by T cells^15,16^. Therapeutic approaches are needed to restore and sustain the T cell responses in ACT, including compounds that might re-express the TIL antigen in melanoma cells. However, the absence of cell-based assay models for drug screening and immune recognition has hampered research in this area.

Towards this goal, we developed a novel cell-based high throughput screening (HTS) assay to identify compounds that would enhance immune recognition in a BRAF mutant melanoma cell model. This *in vitro* two cell assay system measures interferon gamma (IFNγ) as a quantifiable readout which is released after the co-culture of antigen specific CD8^+^ T-cells with melanoma cells. Using this assay, we screened a collection of approximately 500 compounds that was annotated based on targets and pathways and found 36 compounds that increased IFNγ production in the primary screen. Cytotoxic compounds were removed after performing a cell viability assay and hits were further validated using several relevant follow up assays. Finally, four lead compounds were identified which upon confirmation in ACT animal models could serve as a potential adjuvant therapy to ACT treatment.

## Results

### Development of a 1536-well compatible HTS immune recognition melanoma model to measure T cell function

**Figure 1:**
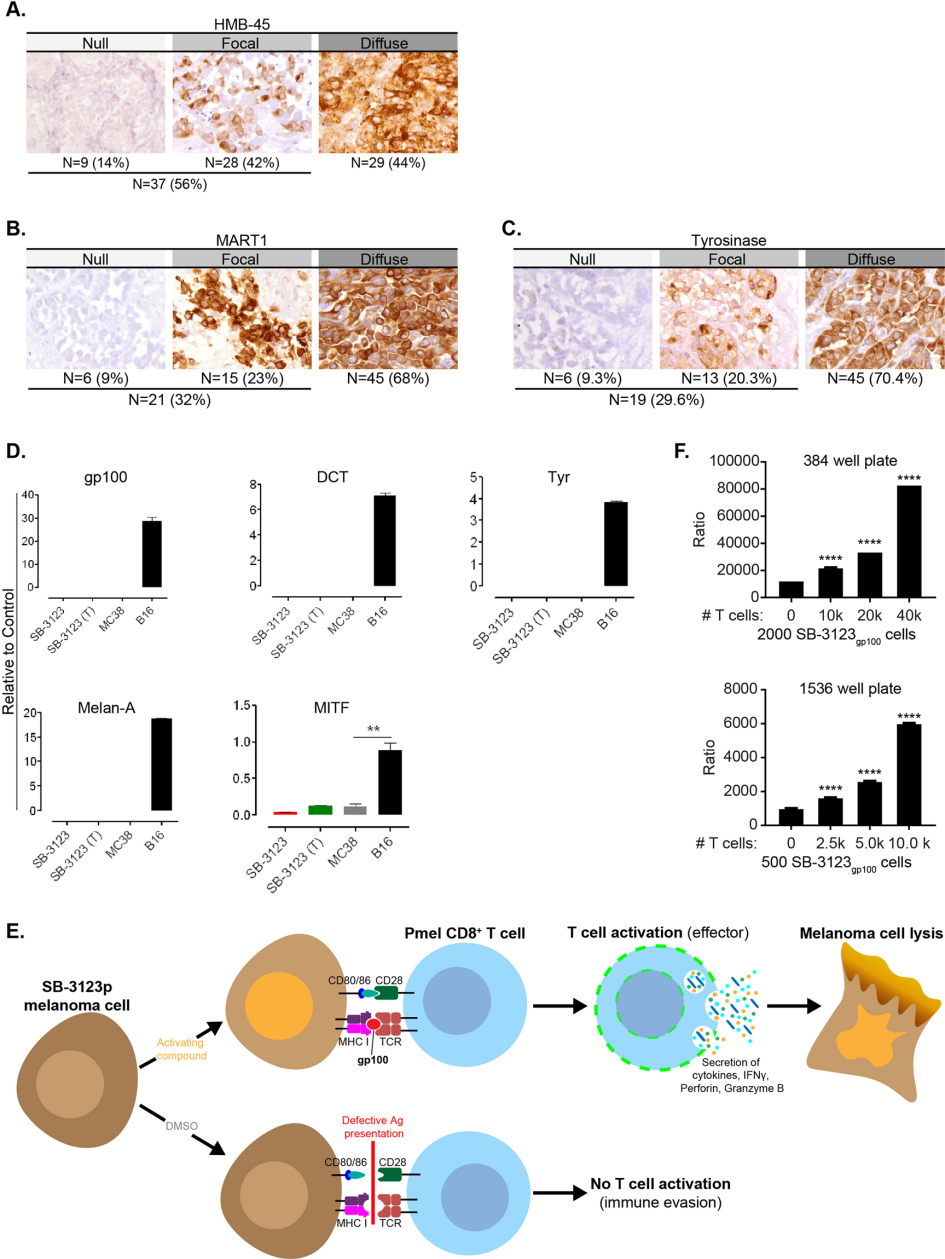
Development of a 1536-well compatible HTS immune recognition melanoma model to measure T cell function (**A**) Immunohistochemistry staining for HMB-45 (gp100) in 5 µm thickness formalin-fixed tissue sections of skin biopsy specimen from human melanoma patients. (**B**) Immunohistochemistry staining for Melan-A (MART1) in 5 µm thickness formalin fixed tissue sections of skin biopsy specimen from human melanoma patients. (**C**) Immunohistochemistry staining for Tyrosinase in 5 µm thickness formalin-fixed tissue sections of skin biopsy sections from human melanoma patients. (**D**) Transcripts for gp100, Dct, Tyr, Melan-A and MITF were quantitated in SB-3123_p_ melanoma cells and in lysate of SB-3123 tumor (T), using qPCR. MC38 colon carcinoma cells were used as negative control whereas B16 melanoma cells were used as positive control. (**E**) gp100 T-cell mediated immune recognition model: Ideal small molecule would up-regulate gp100 on melanoma cells which would be recognized by Pmel-1 CD8^+^ T cells leading to their activation and production of effector cytokines such as IFNγ. (**F**) 2000 SB-3123_gp100_ cells and 500 SB-3123_gp100_ cells were plated in 384 and 1536-well plates and incubated for 48 hours. Varying numbers of ex vivo primed Pmel-1 T cells were added for an additional 24 hours following which IFNγ was quantitated using HTRF kit.

First, we checked if melanoma cells from metastatic melanoma patients expressed tumor antigens that could elicit a T cell response. We analyzed expression of 3 melanoma antigens, gp100 (HMB-45), Melan-A (MART1) and Tyrosinase in skin biopsy samples from metastatic melanoma patients. We observed variability in expression of HMB-45 in patients with metastatic melanoma with 42% patients having focal expression and 44% had a diffuse pattern of staining whereas, 14% patients did not express HMB-45 (Fig. 1A). Similar variability was observed in expression of Melan-A with 23% patients having focal expression, 68% patients with diffuse staining and 9% did not express Melan-A (Fig. 1B). Similarly, 9% of patients did not express tyrosinase whereas, 20% had focal and 70% had diffuse pattern of staining (Fig. 1C). Thus, melanoma patients exhibit heterogeneity in the expression of tumor antigens and there is often absence of tumor antigen expression that may result in immune escape.

We used SB-3123_p_, a genetically relevant *BRAF*^*V600E*^ *PTEN*^*−/−*^ transplantable murine melanoma cell line^17^, to develop our high throughput immune-recognition assay model. We had previously reported that treatment with vemurafenib inhibited the growth of SB-3123_p_ tumors initially, with later developed drug resistance leading to tumor recurrence observed in all mice^17^. We did not detect transcripts for melanoma antigens, gp100, Dopachrome tautomerase (DCT), Tyrosinase (Tyr), Melan-A and detected low levels of expression of Melanocyte Inducing Transcription Factor (MITF) in SB-3123_p_ cells (Fig. 1D). Thus, SB-3123_p_ cells recapitulated key features of human melanoma such as loss of tumor antigen expression and would be an ideal cell line to develop our HTS assay system. As mentioned above, SB-3123_p_ cells do not express gp100 at baseline and we hypothesized that an ideal compound would upregulate gp100 in SB-3123_p_ melanoma cells (Fig. 1E). These melanoma cells would in turn be recognized by T cells from Pmel-1 TCR transgenic mice that recognize the mouse gp100_25–33_ epitope^18^. Enhanced immune recognition would lead to activation of Pmel-1 T cells resulting in acquisition of effector function (increased cytokine production, perforin and Granzyme B secretion) and lysis of melanoma cells (Fig. 1E). Therefore, we utilized IFNγ as a read-out for T cell effector function in our co-culture HTS screening assay model.

We used the SB-3123_gp100_ melanoma cell line retrovirally transduced to express the antigen gp100^17^ as a high signal control for HTS assay development (384-well and 1536-well plate formats). First, we optimized the cell seeding density and found that 2000 (384-well plate) and 500 (1536-well plate) melanoma cells/well allowed us to reach 75% confluence after 48 hours. Next, to establish the co-culture system, we added varying numbers of Pmel-1 T cells to the SB-3123_gp100_ melanoma cells that had been cultured for 48 hours (Fig. 1F). IFNγ was measured 24 hours after addition of T cells using the HTRF IFNγ Assay kit. The increasing number of T-cells resulted in a dose response increase in IFNγ release in both 384-well and 1536-well plate. The assay conditions that produced the maximum signal were a ratio of 1:20 melanoma cells to T cells (500 melanoma cells co-cultured with 10k T cells for the 1536 well plate and 2000 melanoma cells co-cultured with 40 k T cells for the 384 well plate; Fig. 1F). Thus, we developed a co-culture assay system in a high throughput 384 and 1536 well format that can be used to screen compounds that would enhance expression of tumor antigen leading to activation of antigen-specific T cells.

### Screening of a focused collection of mechanistically annotated small molecules using immune recognition-based HTS assay

**Figure 2:**
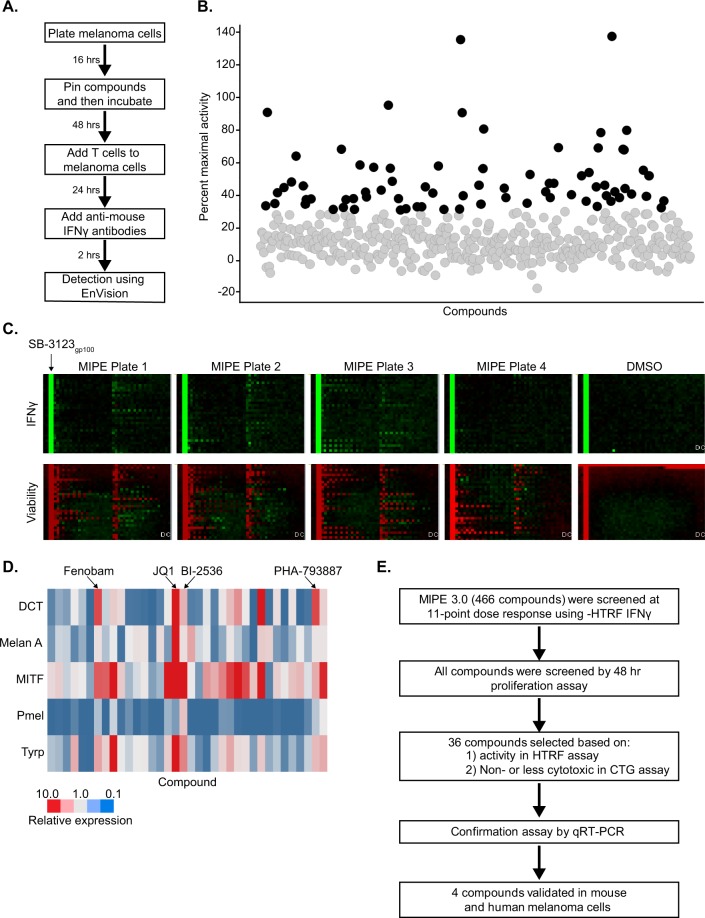
High Throughput Screening of the MIPE 3.0 Oncology Library. (**A**) Protocol for High Throughput Screening Assay. (**B**) The results from the primary screen (IFNγ HTRF assay) displaying maximal IFNγ produced by Pmel-1 T cells on culture with compound treated SB-3123_p_ melanoma cells. (**C**) Top Panel: Heat map for SB-3123_p_ cells (Col 1, 2, 5–48) and SB-3123_gp100_ cells (Col 3, 4). SB-3123_gp100_ cells were used as +ve control and are marked by an arrow in representative 1^st^ plate. For each plate, Col 1 & 2 were treated with DMSO and test compounds were pinned in Col 5–48 and the plates were incubated for 48 hours following which Pmel-1 CD8^+^ T cells were added for another 24 hours. Green depicts an increase in IFNγ quantitated using HTRF. Bottom Panel: Heat map for cell viability assay. SB-3123_p_ cells were plated and pinned with compounds and incubated for 48 hours. For each plate, Col 3 & 4 were pinned with bortezomib (100% death) and Col 1 & 2 were pinned with DMSO (0% death). For each plate, test compounds were pinned in col 5–48 and red depicts a decrease in viability signal or an increase in cytotoxicity. (**D**) Heat map showing gene expression analysis for MITF, DCT, TYRP, Mel A and gp100 in SB-3123_p_ melanoma cells treated with 36 hit compounds at 10 µM concentration. (**E**) Schema depicting the strategy used to select the hits from the HTS screen.

We wanted to screen for small molecules that could be used to enhance immune recognition in a BRAF mutant melanoma cell model. To achieve this goal, we used our co-culture assay system (described above) to screen a focused collection of 466 mechanistically annotated small molecules (NCATS MIPE 3.0). This collection of compounds contains small molecules targeting a broad range of cellular mechanisms and includes chemical probes from the literature, investigational drugs, novel agents entering the clinic, and FDA approved drugs. The assay was performed by plating the SB-3123_p_ melanoma cells in the 1536 well assay plates and allowing them to adhere overnight (Fig. 2A). The next day small molecules in an 11 point concentration-dose response were added to the SB-3123_p_ melanoma cells and the assay plates with melanoma cells and compounds were incubated for 48 hours. *Ex vivo* primed T cells from Pmel-1 transgenic mice were added to the cultures for an additional 24 hours and subsequently, IFNγ was measured using the HTRF assay kit (schema and detailed protocol in Fig. S1A,B). The results from the primary screen (IFNγ HTRF assay) are shown in Fig. 2B (% maximum activity) and Fig. 2C (top panel: heat map of the assay plates). SB-3123_p_ cells treated with DMSO were considered as baseline and SB-3123_gp100_ cells transduced to express gp100 were used as positive control. All compounds were tested at 11 concentrations and concentration response curves for the compounds were generated using the qHTS (quantitative high throughput screening) data analysis paradigm^19^. The concentration response curves were classified in four categories (Class 1, 2, 3 and 4) based on the quality of curve fit to the data (*r*^2^), the magnitude of the response (efficacy), and the number of asymptotes to the calculated curve^19^. All of the plates passed QC (Z′ = 0.86 +/− 0.04 and CV = 4.6 +/− 0.8). We used several criteria to select hits from the primary screen such as the curve class, AC_50_ and the maximum response. Compounds with a curve class of either +1.1, +1.2, +2.1, +2.2 and a maximal response of ≥30% over basal were classified as actives.

In parallel to the IFNγ HTRF assay, a viability assay was done to determine whether in the conditions of the assay, the compounds were cytotoxic (Fig. 2C: bottom panel). SB-3123_p_ cells were treated with compounds, DMSO (100% viability) and bortezomib (0% viability) and incubated for 48 hours following which luminescence was detected after adding Cell Titer Glo cell viability reagent (CTG assay). Some compounds were strongly cytotoxic, some were toxic only at very high concentrations and some were not cytotoxic (Fig. 2C bottom panel). We selected 36 compounds as actives based on their activity in the IFNγ HTRF assay and non-cytotoxicity (Fig. S2A).

It has been shown that melanoma cells are inherently plastic in nature and can switch between differentiated and de-differentiated states leading to loss of antigen and development of resistance^15^. Next, we tested if the 36 active compounds from the IFNγ HTRF assay and the cell viability assay can affect expression of genes that regulate melanoma cells differentiation and pigmentation such as MITF, DCT, Melan-A and Tyrosinase-related protein 1(Tyrp). We also measured if these 36 compounds indeed up-regulate the antigen, Pmel-1 (gp100). An increase in gene expression was seen for many of the genes after compound treatment and 4 compounds that significantly elevated all 5 genes by at least 2-fold were further selected for characterization (Fig. 2D). Figure 2E summarizes the strategy and criteria that were used to select the hit compounds from the drug screen.

### Further validation of the 4 compounds using follow up assays

**Figure 3:**
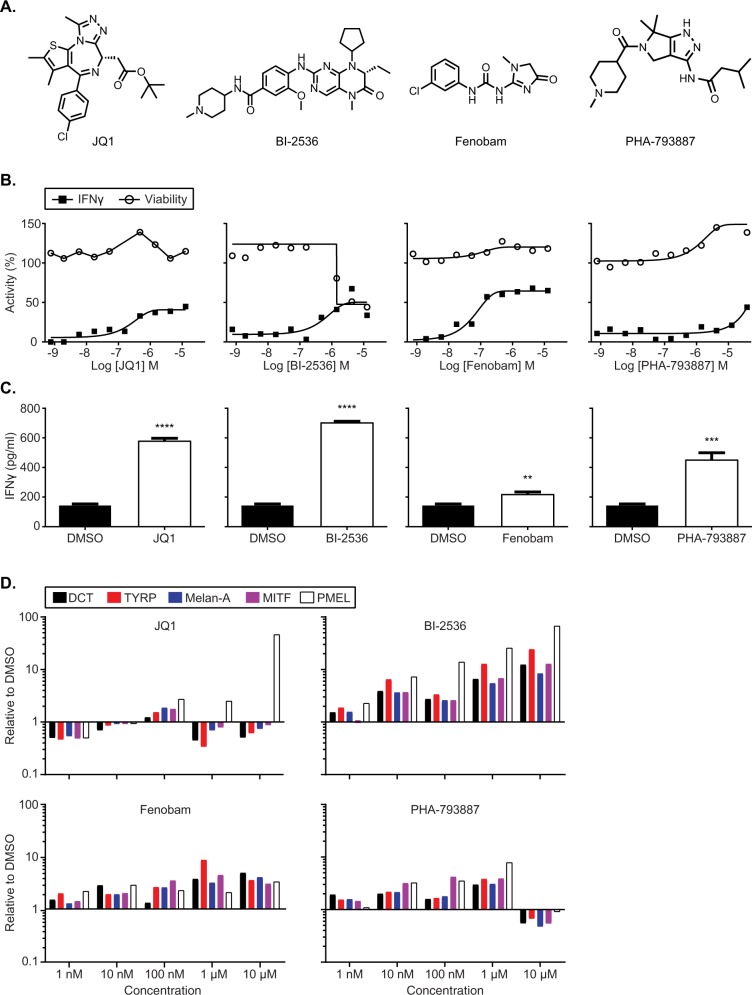
Further validation of the 4 compounds using follow up assays. (**A**) Structure and name of the 4 lead compounds from the HTS screen. (**B**) Dose response curves for IFNγ (IFN HTRF; filled squares) and cell viability (cell titer glo; open circles) for 4 lead compounds with SB-3123_p_ cells plated in 1536 well plates. (**C**) IFNγ quantitated using an ELISA after co-culture of compound treated SB-3123_p_ cells with primed Pmel-1 CD8^+^ T cells in 96 well plates. The plots display treatment of SB-3123_p_ cells with 1.25 µM JQ-1, 0.3 µM BI-2536, 10 µM Fenobam and 10 µM PHA-793887. (**D**) Gene expression analysis for MITF, DCT, Tyrp, Mel A and gp100 in SB3123_p_ melanoma cells treated with 4 lead compounds in a dose response (1 nM, 10 nM, 100 nM and 10 µM).

Based on the activity in the IFNγ HTRF assay, non-cytotoxicity and gene expression analysis, we selected 4 compounds for further follow up studies (Fig. 3A). The four compounds selected have diverse mechanism of action and are as follows: JQ1, a potent and selective BET bromodomain inhibitor^20^; BI-2536, a potent Polo-like kinase 1 (Plk1) inhibitor^21^; Fenobam, a potent and selective metabotropic glutamate receptor (mGluR_5_) antagonist^22^; and PHA-793887, an inhibitor of multiple cyclin dependent kinases (CDK2, CDK5 and CDK7)^23^ (Fig. 3A). Figure 3B demonstrates the concentration dose-response curves with these 4 compounds in the IFNγ HTRF assay and cytotoxicity profiles using the Cell Titer Glo assay. The above mentioned 4 compounds increased IFNγ in a dose-dependent fashion and did not affect cell viability except at very high doses.

Next, we validated the activity of these 4 compounds using a secreted IFNγ ELISA assay in a moderate throughput assay. SB-3123_p_ melanoma cells were plated in 96 well plates and treated with the compounds for 48 hours. *Ex vivo* activated Pmel-1 CD8^+^ T cells were added for an additional 24 hours and IFNγ was measured in the supernatant. As shown in Fig. 3C, JQ1 increased IFNγ (4.5 fold) relative to DMSO, BI-2536 increased IFNγ (5 fold), PHA-793887 increased IFNγ (3 fold) whereas, Fenobam modestly increased IFNγ (1.5 fold) secreted by T cells. Thus, the effect of the 4 compounds on melanoma cells reproduced in an IFNγ secreted ELISA in a 96 well assay format. We also confirmed that the effect of the 4 compounds is primarily via their effect on melanoma cells and not through a direct effect on T cells by culturing the Pmel-1T cells alone (without melanoma cells) with the compounds. Treatment of T cells with the 4 compounds did not increase IFNγ production compared to DMSO treated T cells (data not shown).

We further validated the dose-dependent effect of these compounds on melanoma cells using gene expression analysis. We treated SB-3123_p_ melanoma cells with either JQ1, BI-2536, Fenobam or PHA-793887 and found that the 4 compounds up-regulated the transcription of MITF and its target genes, DCT, Tyrp, Melan A and Pmel-1 in a dose dependent fashion as determined by qPCR (Fig. 3D). Thus, these 4 compounds enhanced expression of melanoma differentiation antigens resulting in enhanced recognition of melanoma cells leading to T cell activation and enhanced effector function as demonstrated by increase in IFNγ production.

### Effects of compounds in human melanoma cells

**Figure 4:**
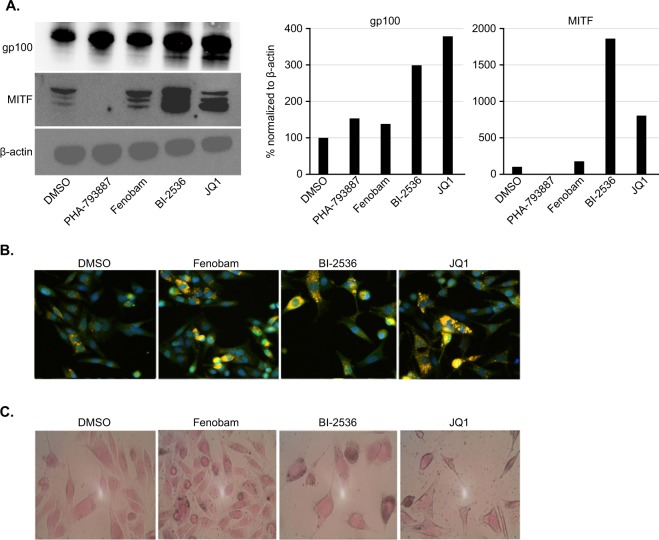
Effect of four lead compounds in human melanoma SK-Mel 28 cells. (**A**) Western blot for gp100 and MITF in SK-Mel 28 human melanoma cells treated with the indicated compounds (PHA-793887 and Fenobam 2.5 µM, PLK1 and JQ1 5 µM). Right panel shows quantitation for gp100 and MITF. (**B**) Immunofluorescence images of SK-Mel 28 human melanoma cells stained for gp100 and MITF 48 hours after compound treatment. Green depicts staining for MITF, orange for gp100 and blue for nuclei. (**C**) Fontana-Masson staining for melanin 96 hours after treatment of melanoma cells.

We have validated the 4 compounds using mouse melanoma cell line, SB-3123_p_ that recapitulates key features of human melanoma^17^. Next, we wanted to extend our studies to a human melanoma model to test if these 4 compounds would also work in human melanoma cells. To this end, we used the mutant BRAF^V600E^ human melanoma cell line SK-Mel 28 similar to mouse SB-3123_p_ cells. First, we treated SK-Mel 28 cells with the 4 lead compounds and among these compounds, JQ1 (4 fold) and BI-2536 (3 fold) increased expression of gp100 protein whereas Fenobam (1.4 fold) and PHA-793887 (1.6 fold) led to a modest increase in gp100 (Figs. 4A and S2B). Next, we interrogated if these compounds would also concurrently increase the master regulator MITF. Treatment with BI-2536 lead to a 18 fold up-regulation of MITF, while JQ1 produced an 8 fold up-regulation. Fenobam increased MITF transcription modestly by 1.8 fold (Fig. 4A). We confirmed these findings using an immunofluorescence assay for gp100 and MITF (Fig. 4B).

An additional follow up study was to look at the ability of the compounds to increase production of melanin pigment, as a marker of melanoma cancer cell differentiation. As depicted in Fig. 4C, treatment with compounds specially Fenobam increased pigmentation in melanoma cells. Thus, these compounds confirmed in a human BRAF mutant melanoma cell model and are promising candidates to be used to potentiate T cell recognition in melanoma patients.

## Discussion

Using a highly immunogenic epitope that can be recognized by a high percentage of melanoma reactive tumor infiltrating lymphocytes^24^, we have developed a 1536 well compatible co-culture cell-based (melanoma cells with immune cells) assay system to identify compounds that can be used synergistically to further enable T cell recognition of melanoma tumor cells. We used this co-culture assay to screen an internal (NCATS owned) collection of ~500 compounds that are known to modulate oncology targets, pathways and phenotypes. Our screening assay is based on the principle of immune recognition of melanoma cells by antigen-specific CD8^+^ T cells. We quantitated IFNγ that was produced by *ex vivo* primed Pmel-1 CD8^+^ T cells on co-culture with melanoma cells that did not express gp100 at baseline but up-regulated gp100 on treatment with compounds. This assay was successfully performed in 1536-well plates using the HTRF IFNγ detection kit and the data correlated well to the screening performed in 384-well plates.

In our assay using BRAF mutant melanoma cells, 4 active compounds were identified that up-regulated the antigen, gp100 and the master regulator MITF. This in turn led to enhanced immune recognition of melanoma cells and an increase in IFNγ produced by the T cells. We have identified and further validated 4 small molecules with diverse mechanism of actions. First, we found that JQ1, a potent inhibitor of the BET family of bromodomain proteins^20^ is a potent up-regulator of melanoma differentiation and hence, enhanced immune recognition. Increasing evidence suggests that epigenetic mechanisms play a central role in pathogenesis of melanoma and a critical role for BRD4 in melanoma tumor maintenance has been reported^25^. Few studies have shown that JQ1 can act synergistically with vemurafenib in BRAF mutant melanoma models^26,27^. Here, our studies demonstrate that JQ1 can also potentiate CD8^+^ T cell recognition leading to enhanced CD8^+^ effector function. We extended our studies to human melanoma cells where treatment with JQ1 potentiated expression of gp100 and MITF. Several BET bromodomain inhibitors are in trial for cancer therapy but the mechanism for the desired effects or unwanted effects such as resistance to therapy are poorly understood^28^. Our assay system provides a unique tool to study mechanistic effects of JQ1 in a co-culture system using both the melanoma cells as well as the immune cells.

We also identified BI-2536, a dual inhibitor of Plk1 and Brd4^21,29^ as a potent up-regulator of gp100. Plk1 is highly expressed in malignant cells including primary and metastatic melanomas and serves as a negative prognostic marker in specific human cancer types^21,30^. In our model, treatment of melanoma cells with BI-2536 led to a huge increase in transcription factor MITF. It would be important to tease apart the biochemical pathways targeted in our model leading to increase in gp100 and MITF and elucidate if the effect on immune recognition was mediated by inhibition of Plk1 or Brd4.

Recently, unbiased proteomics analyses have identified cyclin dependent kinase, CDK2 as a driver of resistance to BRAF inhibitors and MITF expression correlated with CDK2 upregulation in patients^31^. We also identified PHA-793887, a novel and potent inhibitor of CDK2, CDK5 and CDK7^23^ as a compound that affects differentiation of melanoma cells. In our model, cell cycle analysis showed that treatment with PHA-793887 led to fewer melanoma cells in the S phase (Fig. S2C) and hence, cells might stop proliferation and undergo differentiation. These melanoma cells were recognized by the Pmel-1 specific T cells leading to enhanced effector T cell function.

Dysfunction in the glutamatergic signaling pathway has been reported for multiple cancers including melanoma^32^. A phase II trial was conducted in patients with advanced melanoma with riluzole, an inhibitor of Grm1 signaling^33^. The potency and bioavailability of riluzole presented challenges to its clinical use and absorption of riluzole among patients exhibited variability^34^. The mechanism of action of glutamate signaling needs to be interrogated further in metastatic melanoma and our model system could be used to interrogate some of those mechanisms. Our lead compound, Fenobam^22^, a potent and selective negative allosteric modulator of mGluR_5_ also increased pigmentation of melanoma cells and it would be interesting to further explore the mechanisms for the phenotype.

In this study, we have developed a novel HTS cell based co-culture assay to screen for small molecules that can enhance melanoma antigen expression and hence, immune recognition. As a proof of concept, we not only found 4 compounds that augmented IFNγ production in the primary screening assay but also, validated them further using a battery of assays such as toxicity assays, gene expression analysis, Elisa for secreted IFNγ, western blot and immunofluorescence assays for the antigen and tested them in human melanoma cells. These four compounds alone or in synergistic combinations may provide a basis for enhanced immune recognition and may aid design novel therapeutic approaches for patients with BRAF mutant melanoma resistant to immunotherapy.

## Methods

### Cell lines

The SB3123_p_ mouse melanoma cell line was generated by the laboratory of Nicholas Restifo at the National Cancer Institute at NIH. Cells were transferred to NCATS and grown in DMEM (Life Technologies) with 10% heat-inactivated fetal bovine serum (FBS) (Sigma), 1% GlutaMAX (Life Technologies), 1% (v/v) penicillin/streptomycin (Life Technologies), 1% MEM Non-Essential Amino Acids (Life Technologies), 1% Sodium Pyruvate (Life Technologies), 0.1% β-mercaptoethanol (55 mM) (Life Technologies) in 5% CO_2_ at a constant temperature (37 °C) and humidity.

#### SB3123_gp100_ cell line

SB-3123_p_ cells were retrovirally transduced to express mouse gp100^17^. Expression of gp100 was confirmed via qRT-PCR and western blot.

#### SK-Mel 28 cells

Human melanoma SK-Mel 28 cells were bought from ATCC and maintained in Eagle’s Minimum Essential Medium (Life Technologies).

### In Vitro priming of Pmel-1 CD8^+^ T cells

Splenocytes from Pmel-1 transgenic mice were depleted of erythrocytes using ACK lysing buffer and cultured in RPMI containing 10% heat inactivated FBS, 1% glutamine, 1% (v/v) penicillin/streptomycin, 1% Non-Essential Amino Acids, 1% Sodium Pyruvate and 0.1% b-Mercaptoethanol (55 mM). 30 IU/ml human interleukin-2 (NCI BRB) and 1 µm gp100_25–33_ peptide was added and the cells were expanded for 5–7 days.

### Study approval

Pmel-1TCR-transgenic mice were housed in the NIH Clinical Research Center vivarium and maintained in compliance with the National Institutes of Health (NIH) Animal Care and Use Committee. Animal experiments were conducted with the approval of the National Cancer Institute (NCI) Animal Use and Care Committees and performed in accordance with NIH guidelines.

#### Human patient samples

All NIH cancer patients providing biopsies were enrolled in clinical trials approved by the NIH Clinical Center and NCI institutional review boards. All methods were performed in accordance with the relevant guidelines and regulations and each patient had signed an informed consent form.

### Immunohistochemistry

Biopsies were fixed in 10% neutral buffered formalin. 5 µm tissue sections were prepared and placed on poly-L-lysine coated glass slides for routine H&E staining and for immunohistochemistry. The immunohistochemical staining for Mart-1 (Cell Marque, Catalog #: 281M-96, Dilution: 1:100), HMB-45 (ENZO, Catalog #: ENZ-30903, Dilution: 1:4), and Tyrosinase (Leica, Catalog #: NCL-L-TYROS, Dilution: 1:20) was performed on the Roche Ventana Medical Systems BenchMark ULTRA automated IHC platform using the ultraView Universal DAB Detection Kit (Ventana Medical Systems), following standard laboratory protocols established by the histology section of the Laboratory of Pathology at the NIH.

### Mouse interferon-γ homogenous time resolve fluorescence

The day before screening, 500 cells/well in 6 µL were seeded into Greiner-one high base solid bottom white tissue culture treated 1536 well plates (catalog # 789173-F) using a small cassette and a Multidrop Combi dispenser (Thermo Fisher Scientific Inc., Waltham, MA). SB3123_gp100_ cells were also seeded into 2 columns to act as a positive control for the output of interferon-γ release. The plates were allowed to incubate for 16–24 hr at 37 °C, 5% CO_2_, 95% humidity covered with low evaporation stainless steel lids from Kalypsys to allow the cells to adhere. For the generation of standard 11-point dose response curves 23 nL of each dose/compound from the MIPE library was added using a Kalypsys pintool. The plates were incubated for 48 hours at 37 °C, 5% CO_2_, 95% humidity using the same stainless steel lids. After the 48 hour incubation with compounds a total of 10,000 Pmel-1 expressing T-cells (that have been pulsed with 1 μM hgp10025–33 peptide for 5 days) in 2 µL of their growth media were dispensed using a small cassette and a Multidrop Combi dispenser. The plates were incubated for another 24 hours at 37 °C, 5% CO_2_, 95% humidity using the same stainless steel lids. Finally, to detect the amount of IFN-γ, the high range mouse IFNγHTRF kit from CisBio was used. A total 1 µL/well of the HTRF reagent anti-mouse-IFN-γ-d2 diluted in lysis buffer and 1 µL/well HTRF reagent anti-mouse- IFN-γ-K (Eu-Cryptate) in lysis buffer were dispensed using the Flying Reagent Dispenser (FRD) (Aurora Discovery-BD). The plates were incubated for 2 hours at room temperature and then read on the EnVision plate reader using an HTRF protocol (Excitation at 320 nM and Emission at 665 nM for the cAMP-d2 and 615 nM for the anti-cAMP antibody-K). The ratio of 615/615 nM was calculated to normalize for any effects in the donor only channel and those artifacts were removed.

### HTS viability assays

A total of 500 SB3123 cells/well in 5 µL of media were dispensed using a Multidrop Combi dispenser (Thermo Fisher Scientific Inc., Waltham, MA) and a small cassette into barcoded 1536 solid bottom white Greiner One tissue culture treated plates (catalog # 789173-F). For the generation of standard 11-point dose response curves 23 nL of control compound (bortezomib) and library compounds were pinned using a Kalypsys pintool. The plates were then covered with stainless steel cell culture Kalypsys lids and incubated for 48 hours at 37 °C with 5% CO_2_ under 95% humidity and then 3 µL of CellTiter Glo luminescent cell viability assay reagent (Promega) was added using a Bioraptor Flying Reagent Dispenser (Aurora Discovery-BD). The plates were then incubated for 15 minutes at room temperature and the luminescence signal was captured using a 10 second exposure with a ViewLux (Perkin Elmer) containing a luminescent filter. Relative luminescence units (RLU) for each well were normalized to the median RLUs from the DMSO control wells as 100% viability and median RLUs from the bortezomib control wells as 0% viability.

### ELISA for quantitating mouse IFNγ

The day before screening, 2500 SB-3123_p_ melanoma cells/well in 100 µl were seeded into 96 well flat bottom plates. SB3123_gp100_ cells were also seeded to act as a positive control for the output of interferon-γ release. The plates were allowed to incubate for 16–24 hr. The 4 lead compounds were acoustically dispensed in compound plates in a dose response (20 µM, 10 µM, 5 µM, 2.5 µM, 1.25 µM, 0.625 µM and 0.3 µM) and reconstituted in 100 µl media and transferred to the cell plate. The plates were incubated for 48 hours following which ex vivo activated Pmel-1 CD8^+^ T cells were added. Supernatant was collected after 24 hour of co-culture and IFNγ was detected using mouse IFNγ quantikine ELISA kit (R&D systems) following manufacturer’s instructions.

### Mechanism interrogation plate (MIPE) compound library

The library utilized in these studies is an internal collection of 466 high-value small molecules known to modulate oncology targets, pathways and phenotypes referred to as the MIPE-oncology library (MIPE: Mechanism Interrogation PlatE) (a full list of compounds is available upon request).

### Western blot and immunofluorescence

Whole cell lysates for western blots were lysed with RIPA lysis buffer (Thermo Scientific). Proteins were separated by SDS-PAGE, followed by immunoblot analysis using β-actin, and MITF antibodies. The expression of gp100 was detected using PEP13 antisera (gift from Dr Vincent Hearing, National Cancer Institute, NIH, Bethesda). For the immunofluorescence stain, SK-Mel 28 cells were fixed and permeabilized after compound treatment. PEP13 antisera was used to label gp100 and nuclei were stained with Hoechst. Images were read on IN Cell2200 analyzer (GE Lifesciences).

### Fontana masson stain

SB-3123_p_ melanoma cells were grown in 48 well plates and treated with compounds for 96 hours. The cells were fixed and melanin was visualized using fontana-masson stain according to manufacturer’s instructions (Abcam). Briefly, cells were incubated in ammoniacal silver solution followed by incubations in gold chloride, sodium thiosulfate and nuclear fast red solutions.

### Quantitative real time polymerase chain reaction

Once the compounds have been plated using the EDC, 2000 SB-3123_p_ cells were added using a multidrop and cells were incubated for 24 hours. Subsequently, cells were washed with PBS and lysed with cells to signal lysis buffer (Ambion). 10 µl of lysate was transferred to 384 well PCR plate (ThermoScientific clear standard PCR plates AB-1384) and 10 µL RT master mix (Applied Biosystems High-Capacity cDNA Reverse Transcriptase Kit #4368813) was added. The RT step was carried out using AB ViiA 7 LightCycler with the following conditions; Step 1:25 °C for 10 minutes, Step 2: 37 °C for 120 minutes, Step 3: 85 °C for 5 minutes. For the real time quantitative PCR the cDNA was diluted 1:2 in nuclease-free water. The SYBR green master mix (Kapa Biosystems Fast ROX low qPCR reagent KM4118) was then prepared by adding 0.3 µM forward and 0.3 µM reverse primers per reaction. A total of 19 µL SYBR green/primer mix was aliquoted to each well of a new 384-well PCR plate followed by 1 µL of cDNA added to the mixture and the plate covered with clear protective film and briefly centrifuged. The PCR cycling was run as follows: Step 1:95 °C for 15 minutes, Step 2: 94 °C for 15 seconds, 60 °C for 30 seconds and 72 °C for 30 seconds with 40x cycles, and Step 4: melt curve.

A total of five genes of interest and one housekeeping gene were analyzed per sample in triplicate. The mouse gene sequences are as follows:

PMEL-1 (F: CTCTTGTTTCCTGTGGTTCCT and R: GTAGTGGTTCCTTGCCTAGATG)

DCT (F: CCTGTCTCTCCAGAAGTTTGAC and R: CCAGTGTTCCGTCTGCTTTA)

TYRP (F: GAGAGTGGTCTGTGAATCCTTG and R: CTGCTGGTCTCCCTACATTTC)

MITF (F: GAGAAGAGGAGGAAGAGGAAGA and R: CCAGGAAATTGCCGCATTTAG)

18S (F: CACGGACAGGATTGACAGATT and R: GCCAGAGTCTCGTTCGTTATC)

The delta-delta ct was then calculated to compare effects of treatment on gene expression in addition to the melt curve analysis to ensure single product formation.

### Statistics

Data were analyzed using a 2-tailed Student’s t-test and p < 0.05 was considered statistically significant.

## Supplementary information

Supplementary fig 1 and 2.
