# Supplementary material for: Identification of Small Molecule Enhancers of Immunotherapy for Melanoma
Source: Sci Rep. 2020 Mar 30;10:5688. doi: 10.1038/s41598-020-62369-1 (PMC7105471; doi:10.1038/s41598-020-62369-1)
Supplement: Supplementary file 1 — Supplementary fig 1 and 2. [file 41598_2020_62369_MOESM1_ESM.pdf]

Identification of Small Molecule Enhancers of Immunotherapy for Melanoma

Christopher Dextras[1], Myagmarjav Dashnyam[1], Lesley A. Mathews Griner[1], Janani Sundaresan[1], Bryan Chim[2], Zhiya Yu[3], Suman Vodnala[3], Chyi-Chia Richard Lee[3], Xin Hu[1], Noel Southall[1], Juan J. Marugan[1], Ajit Jadhav[1], Nicholas P. Restifo[3], Nicolas Acquavella[3], Marc Ferrer[1*], Anju Singh[1*]

[1]Division of Preclinical Innovation, National Center for Advancing Translational Sciences (NCATS), National Institutes of Health, Rockville, Maryland, USA

[2]Laboratory of Immune System Biology, National Institute of Allergy and Infectious Diseases, NIH, Bethesda, Maryland

[3]Center for Cancer Research, National Cancer Institute (NCI), NIH, Bethesda, Maryland

Figure S1

A.

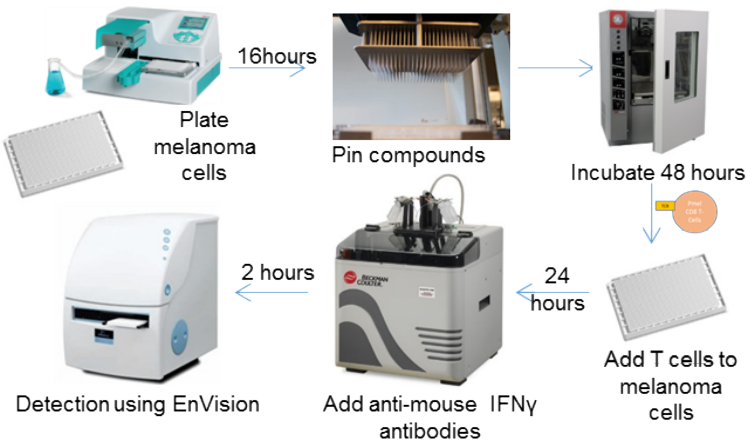

B.

IFN γ HTRF

Both white soild Bottom Tissue Culture Treated Greiner One Plates (# 789173F)

HTRF INFG

Step	Parameter	Value	Description
1	Reagent	6ul	500 cells/well in 6 ul/well SB3123 media multidrop
2	Incubation	24 hours	37C, 5% CO2, 95% humidity
3	Compound	23nl	Columns 1-4 controls, columns 5-48 compounds
4	Incubation	48hr	37C, 5% CO2, 95% humidity
5	Reagent	2ul	10,000 cells PMEL TCells/well in 2 ul/well PMEL media
6	Incubation	24 hours	37C, 5% CO2, 95% humidity
7	Reagent	2ul	HTRF kit: INF-g-d2 and INFg-K in lysis buffer
8	Incubation	2 or 24 hours	Room temperature
9	Detection	Ex 320 nM<br>Em 615 and 665 nM	EnVision plate reader; HTRF mode

Cell Titer Glo

Step	Parameter	Value	Description
1	Reagent	5ul	500 cells/well in 6 ul/well SB3123 media multidrop
2	Incubation	24 hours	37C, 5% CO2, 95% humidity
3	Compound	23nl	Columns 1-4 controls, columns 5-48 compounds
4	Incubation	72 hours	37C, 5% CO2, 95% humidity
5	Reagent	3ul	Cell Titer Glo
6	Incubation	15 minutes	Room temperature
7	Detection	Clear Filter Lum	View Lux

Controls

Columns 1 and 2 DMSO

Columns 3 and 4 Bortezomib, 9.2 uM final

Supplementary Fig

Fig S1

A. Schema for the HTS co-culture assay in 1536 well plates

SB-3123$_{p}$ cells or SB-3123$_{gp100}$ cells were plated and pinned with compounds using pintool. The plates were incubated for 48 hours and thereafter, ex vivo activated Pmel-1 CD8^{+} T cells were added. The plates were incubated for an additional 24 hours following which IFNγ was quantitated using HTRF kit

B: Detailed Protocol for the HTS screening assay

Figure S2

A.

36 Hits demonstrating activation of IFNγ without effecting viability

Sample ID	Sample Name	Curve Class2	AC50(uM)	Max Resp	Efficacy	Inf Activity	Hill Coef	Log AC50	Max Data	Min Data	Primary MOA	Gene Symbol
NCGC00014925-04	Topotecan hydrochloride;Nogitecan hydrochloride/NSC-609699	2.2	5.9508	58.969	57.805	65.572	0.9	-5.2254	58.969	4.322	DNA Topoisomerase I Inhibitors	TOP1
NCGC00022001-07	Picropodophyllin;AXL-1717/NSC-36407	1.2	0.0188	32.142	32.125	33.625	0.5	-7.7254	35.267	4.617	Antimitotic Agent	IGFR1
NCGC00024415-42	Doxorubicin;Adriamycin	2.2	6.6769	72.043	88.667	90.484	0.4	-5.1754	72.043	-0.368	DNA Topoisomerase II Inhibitors	TOP2A
NCGC00025230-01	SB-431542	2.4	13.3221	32.687	42.423	42	0.3	-4.8754	32.687	-5.352	TGF-bR1 (ALK5) Inhibitor	
NCGC00092284-02	AHPN;AGN-192837;AHPN;CD-437	1.2	0.8406	60.824	45.942	54.942	4.955	-6.0754	60.824	5.165	RARgamma Agonist	RARG
NCGC00092384-02	Fenobam;McN-3377	1.4	0.0119	28.983	30.497	31.497	1.1	-7.9254	34.611	2.034	mgluR5 Antagonists	GRM5
NCGC00163411-02	Triptolide	1.2	0.1332	71.12	69.601	80.101	0.7	-6.8754	82.858	7.963	Inhibition of RNA polymerase II?mediated transcription	POLR2A
NCGC00168085-04	Vorinostat;MK-0683;SAHA;Zolinza	1.2	6.1067	46.861	49.298	57.298	0.5	-5.2142	46.861	4.411	Histone Deacetylase (HDAC) 1/2/3/6 Inhibitor	HDAC1
NCGC00185850-02	BIX-01294	2.2	18.818	63.852	74.333	80.129	1.1	-4.7254	63.852	1.818	Histone-lysine N-methyltransferase EHMT2 Inhibitor	EHMT2
NCGC00242495-01	PF-431396;PF-562271	3	29.8245	125.984	151.964	157.984	4.955	-4.5254	125.984	-3.624	Focal Adhesion Kinase (FAK) Inhibitor	PTK2
NCGC00242498-01	D-4476	2.4	23.6905	36.885	44.336	48.336	0.6	-4.6254	36.885	1.261	Casein Kinase I (CK1) Inhibitor	CSNK1A1
NCGC00242506-01	Bortezomib;LDP-341;MLN-341;Velcade	2.1	1.1873	134.172	192.844	177.107	0.3	-5.9254	134.172	-5.546	Proteasome Inhibitor	PSMD1
NCGC00249613-01	Carfilzomib;ONO-7057;PR-171	2.1	1.4948	99.13	101.603	113.603	0.5	-5.8254	99.13	12.815	Proteasome Inhibitor	PSMD1
NCGC00250379-01	CT-99021;CHIR 99021	2.2	16.7716	38.043	46.086	50.086	0.9	-4.7754	38.043	0.736	GSK-3 inhibitor	GSK3B
NCGC00250387-02	KU 0060648	1.2	6.6769	65.378	65.069	71.185	1.464	-5.1754	65.378	1.541	DNA-Dependent Protein Kinase (DNA-PK) Inhibitor	PRKDC
NCGC00250412-01	JQ1	2.2	0.2369	23.021	60.797	54.895	0.4	-6.6254	44.995	-1.463	Bromodomain-Containing Protein 4 (Brd4, HUNK1) Inhibitor	BRD4
NCGC00253438-01	BI-2536	2.2	1.4948	42.202	72.995	82.288	1.21	-5.8254	67.44	3.302	Polo-like Kinase-1 (Plk-1) Inhibitor	PLK1
NCGC00263017-01	CPG-52364	2.3	9.4313	117.137	120.367	127.914	1.66	-5.0254	117.137	1.038	TLR 7, 8 and 9 antagonist	TLR7
NCGC00263091-01	AT-7519	2.2	16.7716	61.396	56.876	62.389	2.406	-4.7754	61.396	1.521	CDK 1 & 2 inhibitor	CDK1
NCGC00263091-02	AT-7519	2.2	21.1142	77.777	77.115	83.999	4.095	-4.6754	77.777	1.617	CDK 1 & 2 inhibitor	CDK1
NCGC00263094-01	AMG-900	2.4	14.9477	45.893	51.958	57.811	0.3	-4.8254	45.893	2.951	Aurora-A/B/C Kinase Inhibitor	AURKA
NCGC00263097-01	Cediranib	2.2	18.818	61.088	76.316	80.475	1	-4.7254	61.088	-1.717	VEGFR-1/2/3 inhibitor	FLT1
NCGC00263129-01	PD-0332991	2.4	21.1142	50.333	59.712	63.339	0.5	-4.6754	50.333	-1.03	CDK4, 6 Inhibitor	CDK4
NCGC00263153-01	AR-42	1.2	0.7492	52.695	44.249	53.749	0.7	-6.1254	52.695	4.482	Histone Deacetylase (HDAC) 1/2 Inhibitor	HDAC1
NCGC00263155-01	Belinostat	2.2	16.7716	60.159	73.346	78.972	0.6	-4.7754	60.159	1.418	Histone Deacetylase (HDAC) 1/2 Inhibitor	HDAC1
NCGC00263166-01	Obatoclax;GX-015-070	2.2	18.818	53.126	61.792	66.688	1.1	-4.7254	53.126	2.089	Bcl-xL inhibitor	BCL2L1
NCGC00263167-01	SNS-032;BMS-387032	2.2	16.7716	73.059	81.912	92.02	1.1	-4.7754	73.059	5.219	CDK2,7,9 Inhibitor	CDK2
NCGC00263168-01	PHA-793887	2.4	16.7716	44.081	49.51	55.463	0.9	-4.7754	44.081	3.139	CDK1,2,3,4,5 Inhibitor	CDK1
NCGC00263173-01	Rucaparib;AG-014699/PF-01367338	2.4	21.1142	53.143	58.648	63.517	0.7	-4.6754	53.143	1.837	PARP-1/PARP-2 Inhibitor	PARP1
NCGC00263182-01	Mocetinostat;MG-0103/MGCD-0103	2.4	18.818	41.194	51.158	53.989	0.6	-4.7254	41.194	-2.088	Histone Deacetylase (HDAC) 1 Inhibitor	HDAC1
NCGC00263191-01	PHA-690509	2.1	11.8734	88.907	84.849	91.218	3.512	-4.9254	88.907	2.594	CDK2/Cyclin A Inhibitor	CDK2
NCGC00263194-01	SB-216763	2.2	3.3464	32.767	37.313	40	0.5	-5.4754	32.767	-2.761	GSK-3 inhibitor	GSK3B
NCGC00263207-01	QS11	2.4	26.5811	43.849	50.979	55.226	0.8	-4.5754	43.849	1.325	ADP-ribosylation factor GTPase activating protein 1 inhibitor	ARF1
NCGC00263238-01	Withaferin A;NSC-101088	2.1	16.7716	87.521	93.942	99.773	1.464	-4.7754	87.521	-4.634	NF-kappaB Activation Inhibitor	NFKB1
NCGC00263265-01	ASR-isobudimer-SO2Ph-4-CH2OC(O)NMe2;ASR-isobudimer-SO2Ph-4-CH2OC(O)NMe2	1.2	2.369	47.269	43.615	51.715	0.6	-5.6254	47.269	5.284	artemesinin analogue	
NCGC00263270-01	Diartallox;BTM-2C-dimer allyl oxime	1.4	0.0749	33.067	32.5	30.5	0.3	-7.1254	33.067	4.3	artemesinin analogue	

B.

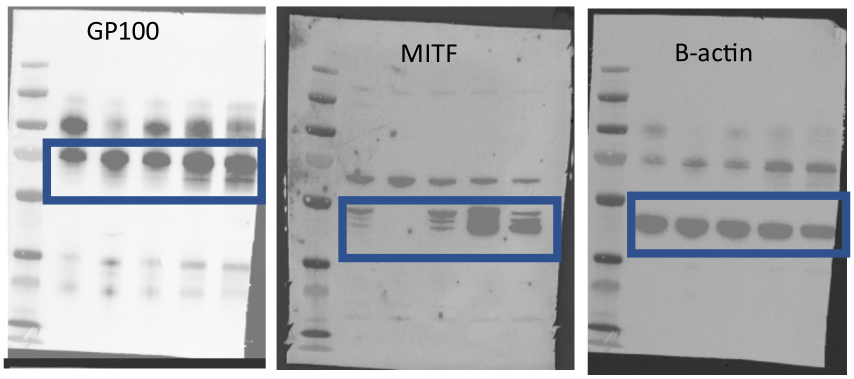

Lane 1: Ladder
Lane 2: DMSO
Lane 3: PHA-793887
Lane 4: Fenobam
Lane 5: BI-2536
Lane 6: JQ1

C.

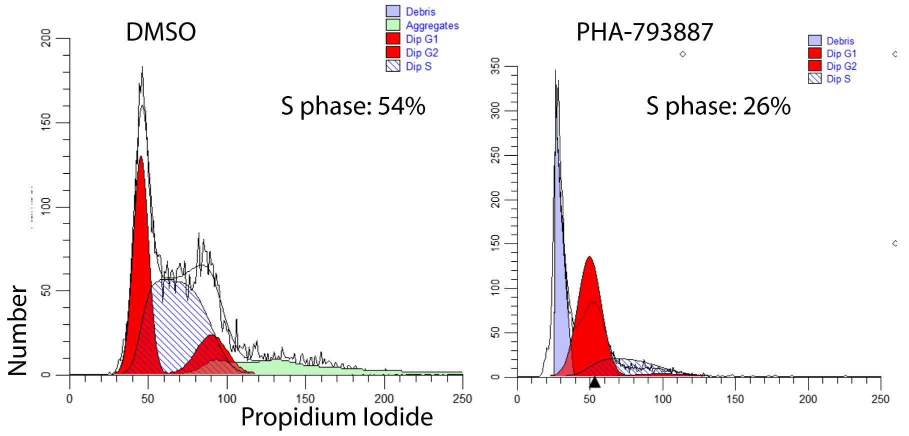

Fig S2

A. List of 36 active hits having an effect in IFNγ HTRF assay without significantly affecting viability

B. Western blot for gp100, MITF and β-actin in SK-Mel 28 cells treated with the indicated compounds.

C. Cell cycle analysis of DMSO or PHA-793887 treated melanoma cells
